# Dorsolateral Prefrontal Transcranial Direct Current Stimulation Modulates Language Processing but Does Not Facilitate Overt Second Language Word Production

**DOI:** 10.3389/fnins.2018.00490

**Published:** 2018-07-25

**Authors:** Narges Radman, Juliane Britz, Karin Buetler, Brendan S. Weekes, Lucas Spierer, Jean-Marie Annoni

**Affiliations:** ^1^Neurology Unit, Section of Medicine, Faculty of Science and Medicine, University of Fribourg, Fribourg, Switzerland; ^2^School of Cognitive Sciences, Institute for Research in Fundamental Sciences, Tehran, Iran; ^3^Leenaards Memory Center, Department of Clinical Neuroscience, Lausanne University Hospital, Lausanne, Switzerland; ^4^Laboratory for Communication Science, Division of Speech and Hearing Sciences, The University of Hong Kong, Pokfulam, Hong Kong; ^5^School of Psychological Sciences, Faculty of Dentistry, Medicine and Health Sciences, The University of Melbourne, Melbourne, VIC, Australia

**Keywords:** tDCS, EEG/ERP, dorsolateral prefrontal cortex, bilingualism, language production

## Abstract

Word retrieval in bilingual speakers partly depends on executive control systems in the left prefrontal cortex – including dorsolateral prefrontal cortex (DLPFC). We tested the hypothesis that DLPFC modulates word production of words specifically in a second language (L2) by measuring the effects of anodal transcranial direct current stimulation (anodal-tDCS) over the DLPFC on picture naming and word translation and on event-related potentials (ERPs) and their sources. Twenty-six bilingual participants with “unbalanced” proficiency in two languages were given 20 min of 1.5 mA anodal or sham tDCS (double-blind stimulation design, counterbalanced stimulation order, 1-week intersession delay). The participants then performed the following tasks: verbal and non-verbal fluency during anodal-tDCS stimulation and first and second language (L1 and L2) picture naming and translation [forward (L1 → L2) and backward (L2 → L1)] immediately after stimulation. The electroencephalogram (EEG) was recorded during picture naming and translation. On the behavioral level, anodal-tDCS had an influence on non-verbal fluency but neither on verbal fluency, nor on picture naming and translation. EEG measures revealed significant interactions between Language and Stimulation on picture naming around 380 ms post-stimulus onset and Translation direction and Stimulation on translation around 530 ms post-stimulus onset. These effects suggest that L2 phonological retrieval and phoneme encoding are spatially and temporally segregated in the brain. We conclude that anodal-tDCS stimulation has an effect at a neural level on phonological processes and, critically, that DLPFC-mediated activation is a constraint on language production specifically in L2.

## Introduction

Learning a second language (L2) appears to be effortless early in life [early bilinguals; age of acquisition (AoA) of L2 before the age of 6–7] but becomes more demanding with age (Fabbro, 2001; Grosjean, 2010). Bilingualism can be defined as the fluent production of more than one language although proficiency in each language can vary. At the neural level, the dominant view is that language representations share the same brain structures, although degree of overlap between languages depends on multiple factors such as AoA, proficiency, degree of immersion in L2, and linguistic (e.g., morphosyntactic) similarity between languages (Kroll and Stewart, 1994; Goral et al., 2007).

Language production is dependent on executive functions (EFs). EFs refer to multiple meta-cognitive abilities that are required to optimize performance when multiple cognitive processes have to be coordinated and comprise processes such as mental shifting, updating, and inhibitory control (IC) of prepotent responses (Miyake et al., 2000). Bilingual language production is particularly dependent on EF, and according to all models of bilingual language processing, verbal fluency involves an interaction between language proficiency and EF. Specifically, EFs are required to control language production in the first and second languages (Postma, 2000; Khateb et al., 2003; Abutalebi et al., 2008; Metuki et al., 2012) because language representations must be monitored both within the language being spoken and between languages to select the appropriate target vocabulary and syntactical construction. In the model of Abutalebi and Green (2016), IC is recruited to avoid interference when the dominant language (L1) competes for access while speaking the non-dominant second language (L2; Green, 1998; Sunderman and Kroll, 2006).

Bilateral prefrontal cortices, and most notably the dorsolateral prefrontal cortex (DLPFC) and the dorsal anterior cingulate cortex (dACC; MacDonald et al., 2000; Koechlin et al., 2003), are assumed to be crucially involved in EFs. DLPFC is mostly involved in selecting a target response and suppression of inappropriate response and the manipulation of information held in working memory (Rowe et al., 2000), while ACC is involved in conflict monitoring and error detection (Botvinick et al., 2004). For example, patients with lesions in prefrontal cortex show reduced verbal fluency, which in the most extreme cases can resemble mutism (Kolb and Taylor, 1981; Coelho, 1995). Thus, Abutalebi and Green (2016) assume that the brain regions activated during EFs are also recruited during language production for fluent bilingual speakers (Price, 2000, 2010).

Language production in bilingual speakers have been measured at the word (lexeme) level on picture naming and word translation tasks which put differential requirements on EF: relatively low in picture naming and relatively high in word translation. Models of picture naming assume that word retrieval requires a number of processing stages: visual attentional control, access to semantic representations (from 0 to 200 ms after image presentation; Noppeney et al., 2004; Whitney et al., 2012), lexeme selection (to 275 ms post-picture presentation), speech monitoring and inhibition of lexical competitors followed by phonological code retrieval (at around 350 ms), syllabification (at around 450 ms), and phonetic encoding (motor programming of individual syllables) at around 600 ms which precedes articulation (Indefrey and Levelt, 2004; Indefrey, 2011).

The Revised Hierarchical Model (RHM) assumes a separate lexicon for each language (Kroll and Stewart, 1994; Kroll et al., 2010). These separate lexica are linked to a single semantic or conceptual system, which contains word meaning representations. The RHM assumes asymmetry in the strength of the connections between words and concepts in two languages, thereby postulating that associations between lexical nodes and concepts are weaker in a less proficient language (Stein et al., 2009). Therefore, in low proficiency bilinguals, access to L2 is assumed to require mediation *via* translation of L1 equivalents, which in turn leads to a processing cost and therefore slower responses. According to the RHM, forward translation (from L1 to L2) will be slower compared to backward translation (from L2 to L1) for bilinguals who are less proficient due to the later acquisition of L2 after early childhood or when L2 is the less dominant language (Kroll and Stewart, 1994; Sholl et al., 1995); importantly, this model does not claim the importance of EF for the difference in L1 and L2 processing.

Abutalebi and Green’s IC model (Green, 1998; Abutalebi and Green, 2016) offers an alternative explanation for the translation asymmetry effects. According to their model, forward translation requires inhibition of L1 lexical nodes to allow spoken production of L2 words. For bilinguals who have “unbalanced” proficiency in two languages, the lexical nodes in L2 are assumed to be less active than lexical nodes in L1. This requires additional attentional resources for processing the L1 lexical nodes, i.e., for redundant nodes to be suppressed. Consequently, forward translation is slower when compared to backward translation because the tasks require differential inhibitory demands (Price, 2000; Sunderman and Kroll, 2006; Kroll et al., 2010).

Behavioral and fMRI studies report significant associations between neural activity in the prefrontal cortex and performance on picture naming and word translation tasks (Klein et al., 1995; Price, 2000; Khateb et al., 2007). However, it is not clear whether the DLPFC is necessary for word retrieval in these tasks. FMRI studies report correlational information. Most relevant to neurocognitive models of bilingualism is the putative role of the DLPFC in cognitive control within a language (e.g., naming in L1 or L2) vs. between languages (e.g., translation). Current evidence for a causal role of the DLPFC in cognitive control during language processing in bilingual speakers is restricted to lesion studies, which can only confirm whether the DLPFC is sufficient for language control and not whether it is a necessary mechanism. Transcranial direct current stimulation (tDCS) is a safe, reliable, transient, and effective method to study the necessary and sufficient conditions for different cognitive functions such as language processing (Nitsche et al., 2008). tDCS is assumed to induce changes in resting potentials of neurons and these appear to be polarity specific, that is, anodal-tDCS increases and cathodal-tDCS decreases cortical excitability (Nitsche et al., 2003). Although there is a general trend for anodal-tDCS to have a facilitating effect on cognitive performance, there are mixed results on effects of anodal-tDCS over DLPFC on picture naming in monolingual speakers (Horvath et al., 2015). These controversies resonate with meta-analyses on the effect of tDCS over DLPFC on working memory tasks, namely, improvement in reaction times but not accuracy (Brunoni and Vanderhasselt, 2014; Dedoncker et al., 2016; Hill et al., 2016). In addition, a meta-analysis investigating the effect of tDCS over DLPFC on different cognitive functions (EFs, language, and memory) showed no effect of anodal-tDCS on performance (Horvath et al., 2015). In their review, Horvath et al. (2015) argue that the lack of tDCS-induced behavioral effects can be due to inter-subject differences and that the lack of detailed data (especially for null-effects) makes it difficult to explain the absence of effects. They also argue that tDCS is a weak form of stimulation that might not be able to modulate the functioning of the healthy brain. Anodal-tDCS has been used over the DLPFC to modulate domain-general and language-specific cognitive control (Fertonani et al., 2010; Jeon and Han, 2012; Metuki et al., 2012; Horvath et al., 2015; Hussey et al., 2015; Hill et al., 2016) but it has not yet been used in bilingual language processing.

According to the IC model, the DLPFC constrains performance in language production. The goal of this study is to investigate whether anodal-tDCS over the left DLPFC improves language production in bilingual speakers. Following the argument by Horvath et al. (2015), stating the relatively weak modulation of the healthy brain, to overcome the limitations of discrete behavioral measures, we recorded the electroencephalogram (EEG) during bilingual word production (picture naming in L1 and L2 and backward and forward translation) to assess putative effects of anodal-tDCS over DLPFC on the brain processes underlying L1 and L2 production. We tested the specific hypothesis that increasing brain activity of cognitive control areas could modify networks implicated in lexical search and selection during word production in L2 but not in L1. In addition to picture naming and translation tasks, we used a non-verbal fluency task as a measure of domain general EF, and a phonemic verbal fluency task as a measure of lexical access and EF (Shao et al., 2014). Based on the RHM, the relationship between lexica and the conceptual system depends on language proficiency which is weaker in the less proficient language (Kroll and Stewart, 1994; Kroll et al., 2010). According to the IC model (Green, 1998), less proficient bilinguals rely more on strategic control during language production in L2 (Hernandez et al., 2005). As L2 proficiency increases, the lexico-semantic processing of L2 target words is less dependent on the activation of executive control and therefore the prefrontal cortex (Stein et al., 2009). Critically, the IC model assumes that cognitive control in language production is not domain (language) specific. Following these assumptions (Hernandez et al., 2005; Kroll et al., 2010), we therefore hypothesized that anodal-tDCS stimulation over the left DLPFC would enhance behavioral performance on both verbal and non-verbal fluency tasks.

The RHM and IC models offer complementary explanations why picture naming is more difficult in L2 than L1 and why forward translation is more difficult than backward translation. But only the IC model assumes a role of EF in this process. Hence according to the IC but not the RHM model, anodal-tDCS should improve performance that relies more on EF. Therefore, improvements should be reflected maximally in improved response accuracy on verbal fluency tasks in L2, and response accuracy and reaction time in picture naming in L2 and word translation during forward translation (which is more demanding on the control system and requires activating lexical nodes in L2 while inhibiting lexical nodes in dominant L1). At the level of EEG activity, stimulation should impact on the time windows related to executive functioning. These effects should be observed in early pre-lexical attentional processes including language switching, language selection, and target lexical selection (>250 ms post-stimulus in naming and ∼400 ms post-stimulus in translation) according to the results reported by Christoffels et al. (2013).

## Materials and Methods

### Participants

We recruited native French speakers who acquired English (L2) as a second language after 7 years of age. All were all right-handed according to the Edinburgh Handedness Inventory (Oldfield, 1971) with no history of neurological, psychological, or other health problems. We recruited initially a total of 28 participants. However, for verbal and non-verbal fluency task, only 26 participants (mean age: 24.3 ± 6.4, *N* = 9 males) were included (one participant was excluded due to very low L2 proficiency and another was excluded due to missing data error). For the picture naming and translation tasks, 24 participants (mean age: 24.5 ± 6.5, *n* = 8 males) were included (three of the initially recruited participants were excluded because of missing data and one due to very low L2 proficiency).

### Second Language Proficiency

#### Age of Acquisition and Immersion

Age of acquisition, immersion, and self-evaluation of language use was assessed using a custom-made questionnaire (Radman et al., 2016). We assessed immersion in French and English by asking the following information: the AoA, how long they lived in a region where the dominant spoken language was English or French, which language they spoke with their family members, in school, in present activities (watching TV/listening to radio, reading books, arithmetic), and if the language was acquired in school or out of school only. In the same questionnaire, participants were asked to indicate in percentage terms how well they would estimate their English reading, speaking, comprehension, and writing skills (**Tables 1, 2**). As expected, greater usage and immersion of French was reported compared to English.

**Table 1:** Age of acquisition and L2 proficiency of participants (n = 24): using a questionnaire, participants were asked to indicate in percentage terms how well they would estimate their English reading, speaking, comprehension, and writing skills.

Variable	Mean	*SD*
Age of acquisition (years)	12.62	2.6
**L2 Self-evaluation (%)**		
Speaking	60.5	15.5
Comprehension	70.5	13.8
Reading	75.5	12.2
Writing	57.6	20.6
**L2 Vocabulary Tests**		
DIALANG L2 vocabulary score (minimum = 0; maximum = 1000)	656.6	156.6

*The vocabulary subtest from the computer-based DIALANG language diagnosis system was administered to evaluate vocabulary knowledge in English.*

**Table 2:** L1 and L2 immersion and usage of participants (*n* = 24).

	Language usage	Mean	*SD*
**L2 immersion and use**	Use at work/studies (%)	19.8	17.6
	TV/radio (%)	27.2	23.2
	With friends (%)	11	12
	Reading books (%)	25	24
**L1 immersion and use**	Use at work/studies (%)	80.21	17.6
	TV/radio (%)	73.95	23.36
	With friends (%)	89.58	12.32
	Reading books (%)	75	23.93

#### Computer-Based Vocabulary Knowledge Evaluation in English

The vocabulary subtest was taken from the computer-based DIALANG language diagnosis system and administered to evaluate vocabulary knowledge in English (Zhang and Thompson, 2004). In this test, participants indicate for each of 75 stimuli whether it is a correct word in English or a highly word-like pseudo-word. The mean DIALANG score in English vocabulary was 656 ± 159 in our participants, confirming an intermediate level of English vocabulary. Information on L2 skills is summarized in **Table 1**.

### Stimuli and Task Procedure

#### Online Tasks

##### Verbal fluency task

Participants were asked to produce words starting with a specific letter within 1 min (we used letter “P” in L1 and letter “S” in L2). The selected letters have been used in previous studies with healthy and clinical populations in both languages (Paradis and Libben, 1987; Tombaugh et al., 1999). “P” and “S” are considered to be “easy letters” (Borkowski et al., 1967). Participants were instructed to produce any word except for proper nouns, repetitions, and verb conjugations. Scoring was based on the number of words produced.

##### Non-verbal fluency task

Participants were given a fixed configuration of dots and asked to generate as many novel designs as possible in 120 s while avoiding repetitions and other rule-breaks (Cattelani et al., 2011). Blind scoring was based on the number of unique designs produced and the percentage of perseverative errors [(perseverative errors/total unique designs) × 100; Fernandez et al., 2009].

#### Offline Tasks

##### Picture naming

**FIGURE 1:**
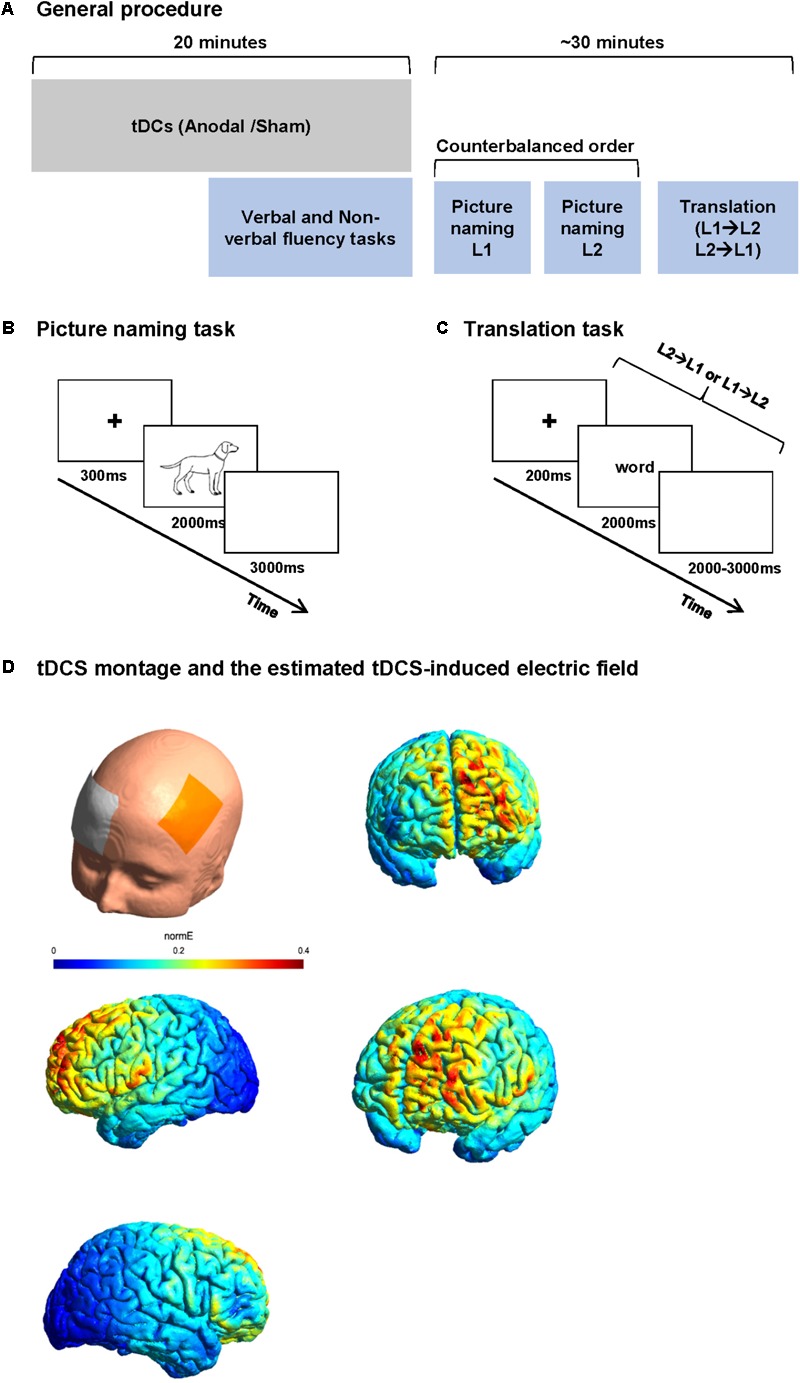
Study procedure and tasks. **(A)** Study procedure: each participant participated in two sessions 1 week apart. In each session, participants received 20 min of anodal or sham tDCS. The participants performed verbal and non-verbal fluency task before the end of stimulation. After the stimulation, the participants performed two blocks of picture naming (in L1 and L2 in a counterbalanced order between subjects) and finally a word translation task. **(B)** Picture naming paradigm. Participants were asked to name the images presented on the screen as fast as possible. **(C)** Word translation task. Participants were randomly presented words in L1 or L2 and were asked to translate it to L2 or L1 as fast as possible. **(D)** An estimate of the electric field induced by our tDCS montage in the anodal condition.

Stimuli comprised two series of 70 pictures to be named in French (list 1) and English (list 2). Items were selected from the Snodgrass image corpus (Snodgrass and Vanderwart, 1980). Selected names were non-cognates. Word frequency, name agreement, and image agreement were matched across lists based on data reported by Alario and Ferrand (1999). French words were longer than English words (mean letter count 6.24 vs. 5.16, *t* = 3.47, *p* < 0.001). Image familiarity was matched across list. Image complexity [rating of the detail/intricacies of the line drawing (Snodgrass and Vanderwart, 1980)] was higher for the French list (average complexity index was 3.07 for French and 2.71 for English lists, *t* = 2.49, *p* = 0.01). All images consisted of line drawings of approximately the same size (no larger than 540 × 400 pixels) with a consistent white background. Participants were instructed to name pictures as quickly as possible. Stimuli were presented for 2000 ms at the center of a 15′ LCD screen with a refresh rate of 60 Hz. Each picture was preceded by a fixation cross for 300 ms. A white color screen of 3000 ms followed the picture. A short training was performed for the tasks during tDCS stimulation. The training consisted of series of five trials for naming and translation (different from the stimuli of the study), which were the same for all participants. The training was performed in both sessions (**Figure 1B**).

##### Word translation

Stimuli consisted of two lists of five- to eight-letter concrete nouns; List 1 contained 50 French words to be translated in English (forward translation) and List 2 contained 50 English words to be translated in French (backward translation). Items were selected from Wordgen (Duyck et al., 2004) and CELEX (Baayen et al., 1996) databases. None of the lists contained cognate words. The lists were matched for word length, frequency, and neighborhood size based on Wordgen and CELEX databases. Each word was presented on a 15′ LCD screen with a refresh rate of 60 Hz for 2000 ms preceded by a fixation cross of 200 ms and followed by a blank screen of 2000–3000 ms. Stimuli in both languages were presented in the same block in a random order. Participants were instructed to translate the presented word as fast as possible in the other language. No cue was given about the direction of translation (**Figure 1C**).

For the picture naming and translation tasks, stimulus presentation and responses were recorded using a voice activated key controlled using E-Prime 2.0 (Psychology Tools, Inc., Pittsburgh, PA, United States). In these tasks, only the first oral responses given after stimulus onset were considered. Synonymous words (in the picture naming task according to Snodgrass normative data and in word translation task according to Merriam-Webster’s dictionary) were also considered as correct responses.

### Study Design

Each participant participated in two sessions presented 1 week apart. In each session, participants received 20 min of anodal or sham tDCS. Because the effect of tDCS is known to reach the maximum at around 4 min after the start of stimulation (Nitsche et al., 2003, 2007, 2008), we performed verbal phonemic (in L1 and L2) and non-verbal fluency tasks (a modified five points test, as a control task) in a controlled order (1 – verbal fluency in L1, 2 – non-verbal fluency, and 3 – verbal fluency in L2) starting 6 min before the end of stimulation (online). After anodal-tDCS stimulation, participants were asked to perform picture naming and word translation tasks while EEG was recorded simultaneously (**Figure 1**). The order of stimulation type was counterbalanced between the participants.

After preparing the tDCS electrode montage [active electrode over F3 and reference electrode over the right supraorbital area, cf. Section “Transcranial Direct Current Stimulation (tDCS)”], an EEG cap was placed on the head, over the tDCS electrodes. In order to minimize the time interval between the end of stimulation and the beginning of the tasks, the EEG electrodes were already attached before the stimulation (except for the EEG electrodes of the frontal part because of the overlap between tDCS and EEG electrodes). Each participant then received anodal or sham tDCS. After the end of stimulation, the tDCS electrodes were removed and the EEG montage was completed. Participants were then asked to perform three tasks while EEG was recorded. The tasks started with two blocks of picture naming [one block in L1 and one in L2 (counterbalanced order across participants)] and one block of word translation (mixed forward and backward) always in the last part of the session. The ensemble of three language tasks was designed to last maximally 25 min to ensure the stability of tDCS after effect (Nitsche et al., 2008; **Figure 1A**).

The protocol was first evaluated in a pilot study to confirm the feasibility of EEG recording during the stability of tDCS effects (i.e., considering the time limit of tDCS after-effect and the effect of the wet area of the tDCS on subsequent EEG recording). Our pilot study confirmed that the planned EEG recording after tDCS could indeed be conducted during the expected tDCS after-effect. EEG recording was not affected by the wet area as during EEG data preprocessing, only limited bridges between electrodes was found as a result of the wet area (see section “EEG Acquisition and Preprocessing”).

At the beginning of the first session, participants filled in the questionnaires on their health status, handedness, and their second language proficiency (AoA, immersion, and self-evaluation). They also performed a vocabulary subtest using the DIALANG language diagnosis system (for more details, see section “Second Language Proficiency”). At the end of each session, participants were asked to fill in a questionnaire on tDCS side effects that they might have noticed during that session.

This study was carried out in accordance with the recommendations of “Swiss Ethics Committees on research involving humans” with written informed consent from all subjects. All subjects gave written informed consent in accordance with the Declaration of Helsinki. The protocol was approved by Cantonal Ethics Committee of Vaud. This study was also registered as a clinical trial in clinical trial registry of the U.S. National Institutes of Health^1^ (ID: NCT02289521).

### Transcranial Direct Current Stimulation (tDCS)

#### Region of Interest

Stimulation was performed using DC-STIMULATOR PLUS (Eldith, neuroConn GmbH, Germany) through a pair of rubber iso-potential electrodes fully enclosed in saline-soaked sponge pocket over the left DLPFC using tDCS. We used isotonic (0.9%) NaCl saline solution. The active electrode was placed over F3 of a 10–20 EEG system. F3 has been constantly used to target DLPFC (Nitsche et al., 2009). To find the F3 accurately, we used the system introduced by Beam et al. (2009) and confirmed the point with an EEG cap. The return electrode was placed over the right supraorbital area.

#### Electrode Size

To increase the focality of stimulation under the active electrode and decrease the effect of stimulation under the return electrode, we selected different sizes of electrodes (Nitsche et al., 2007, 2008): the smaller electrode (7 cm × 5 cm) as active and the larger electrode (7 cm × 10 cm) as return electrode. The sponge–electrode–sponge thickness was 2–1.9–2 mm.

**Figure 1D** shows an estimate of the electric field induced by our tDCS montage in the anodal condition. The estimation of the distribution of the electric field was generated in SimNIBS 2.0.1 (Thielscher et al., 2015). The model is based on the following conductivity values for its key anatomical components [SimNIBS default values, as in, e.g., Thielscher et al., 2015 or de Lara et al., 2017: scalp (σ = 0.465 S/m), bone (σ = 0.010 S/m), cerebrospinal fluid (σ = 1.654 S/m), gray matter (σ = 0.275 S/m), and white matter (σ = 0.126 S/m)]. The volume mesh and visualization were generated through Gmsh (Geuzaine and Remacle, 2009).

#### Duration and Current Density

A constant current of 1.5 mA was applied for 20 min (fade in: 15 s and fade out: 20 s) for anodal-tDCS resulting in a current density of 0.042 mA/cm^2^ under the active electrode. This stimulation duration is suggested to result in a shift in cortical excitability up to 60 min (Nitsche et al., 2008). In the sham condition, there was a short direct current of 30 s (8 s fade in and 5 s fade out) at the beginning of the stimulation. There was no direct current stimulation after the first 43 s, except for small pulses of 3 ms every 550 ms emitted by the stimulator for impedance checking. The average current over time is not more than 2 μA. This way, the participant is less able to detect any difference between sham and real stimulation (Neurocare Group, 2013).

### Analysis of Behavioral Data

#### Online Tasks

Scores on the verbal fluency task were analyzed using a 2 × 2 within subject design with factors Language (L1; L2) and Stimulation (anodal; sham) in a two-way repeated measures ANOVA. Additionally, we have performed this analysis while controlling for the DIALANG score (as the measure of L2 proficiency), implemented in the model as a covariate. For the non-verbal fluency task, a paired *t*-test compared scores of the number of unique designs as well as perseverative errors between anodal and sham tDCS.

#### Offline Tasks

Response accuracy and voice onset time (VOT) on the picture naming task were implemented in a 2 × 2 within subject design with factors Language (L1; L2) and Stimulation (anodal; sham; using two-way repeated measures ANOVA). We have also performed this analysis while controlling for the DIALANG score (as the measure of L2 proficiency). The behavioral data from the translation task (response accuracy and VOT) were subjected to a 2 × 2 within subject design with factors Translation direction (Forward; Backward) and Stimulation (anodal; sham). We have also performed this analysis while controlling for the DIALANG score (as the measure of L2 proficiency). Behavioral analyses were performed using SPSS 21.

#### Comparison of tDCS Effects Across Online and Offline tasks

Horvath et al. (2015) have put forth two explanations for the lack of behavioral effects of tDCS in cognitive tasks. They argue that (i) tDCS is not strong enough to produce an effect or that (ii) inter-individual differences obliterate the effects. If the latter is true, such inter-individual differences should be consistent across tasks. We hence assessed the consistency of tDCS across online and offline tasks and their relation to the DIALANG vocabulary score. To that end, we computed the effects of tDCS in the verbal and non-verbal fluency tasks and on the VOT and error rates for naming in L1 and L2 and backward and forward translation by subtracting the respective values in the sham from those of the anodal stimulation condition. Next, we computed correlation coefficients between those values and the DIALANG vocabulary score and corrected the so-obtained *p*-values using the False Discovery Rate (Benjamini and Hochberg, 1995).

### EEG Acquisition and Preprocessing

Electroencephalogram was sampled continuously at 1024 Hz from 64 preamplified Ag/AgCl electrodes using an ActiveTwo system (Biosemi, Inc., Amsterdam, Netherlands). Electrodes were placed in an elastic cap according to the extended 10–10 system, the reference electrode was placed at the vertex (“Cz”), and impedances were kept below 10 kΩ. Electrode preparation was done with SignaGel (Parker Laboratories, Inc., Fairfield, NJ, United States), in order to use the same electrolyte (NaCl) for EEG and tCDS electrodes. This was done to avoid the buildup of low frequency battery potentials that can arise from mixing different electrolytes which can affect EEG recordings. Offline, the data were recomputed to average reference and digitally band-pass filtered between 0.18 and 40 Hz using second-order Butterworth filter with a -12 db/octave roll-off. The filter was computed linearly with two passes (one forward and one backward), eliminating the phase shift, and with poles calculated each time to the desired cut-off frequency. This was done using epochs spanning -500 to +500 ms around the selected epochs. Additionally, a notch filter (50 Hz) was used to remove AC noise.

All offline analyses were performed using the Cartool software by Denis Brunet^2^ and the STEN toolbox developed by Jean-François Knebel.^3^ To avoid possible topographic distortions caused by electrodes bridges (especially because of a possibly wet skin over frontal area caused by tDCS electrodes), the eBridge toolbox in EEGLAB was used to find any possible bridges in the raw EEG files (Alschuler et al., 2014).

Epochs from 100 ms before the presentation of the stimuli to 600 ms (for picture naming task) and to 800 ms (for word translation task) after the stimulus onset were extracted for each condition. Data were baseline corrected over the whole epoch at single epoch level. Event-related potentials (ERPs) were calculated by averaging the extracted epochs for each participant and condition separately (i.e., in picture naming task: picture naming in L1 and L2 following anodal and sham stimulation and in word translation task: forward and backward translation following anodal and sham stimulation). Only trials with correct responses were included. Epochs with eye-blink or other artifacts (as determined by amplitude changes exceeding 80 μV on at least one electrode during an epoch) were rejected before averaging (Lee et al., 1999). Since longer epochs were extracted for the translation task, more epochs were contaminated with eye-blinks. This resulted in too many trials being removed because of eye-blink. Therefore, for the translation task, independent component analysis (ICA) was implemented using the extended *runica* algorithm in EEGLAB to remove eye-blinks. This led to increase the number of trials included in the ERP. Before group averaging, electrodes exhibiting substantial artifacts as well as bridged electrodes from each participant were interpolated using a three-dimensional spline algorithm before statistical analyses (mean 6.25% interpolated electrodes; Perrin et al., 1987). There was on average 1.25 (±1.3) EEG sensor bridges per participant. Nine subjects showed no EEG sensor bridges in both the two sessions.

### Electrical Neuroimaging Data Analysis

#### Analysis of ERP Waveforms

Electrode and time-point wise analysis of the ERPs was conducted by comparing the ERPs to L1/Anodal, L1/Sham, L2/Anodal, and L2/Sham for picture naming using a 2 × 2 within-subjects ANOVA with factors Language and Stimulation for word translation at each scalp electrode as a function of peri-stimulus time. The same analysis was performed for ERPs to Forward/Anodal; Forward/Sham; Backward/Anodal; and Backward/Sham conditions for word translation with factors Translation direction (Forward; Backward) × Stimulation (Anodal; Sham). This data-driven analysis allows us to identify peri-stimulus time windows showing sustained effects without being limited to *a priori* selected time windows (e.g., specific ERP components). Correction was made for temporal and spatial auto-correlation through the application of a >11 contiguous data points (10 ms) on at least 10% of electrodes criterion for the persistence of significant effects (Guthrie and Buchwald, 1991). The analysis was performed using the STEN toolbox.

For the periods of interaction between the studied factors, to identify the direction of the effects, sources estimations were computed and statistically analyzed. In fact, in contrast to the source analyses, waveform analyses depend on the selection of the electrode. The direction of the effects found at waveform level is not necessarily similar to the results obtained from statistical analyses on source level. We therefore interpret the results based on the statistical analyses at source level. The details on intracranial source estimation and analyses come in the next part.

#### Analysis of Intracranial Sources and Source Differences

The estimation of intracranial generators for a given scalp topography is an ill-posed problem, because a given scalp topography can in principle be generated by any combination of intracranial sources (inverse problem). This inverse problem can be overcome by implementing known biophysical constraints of the generation and propagation of intracranial sources. We used a distributed linear inverse solution based on local autoregressive average (LAURA) regularization approach (Grave de Peralta Menendez et al., 2001; Michel et al., 2004). LAURA selects the source configuration that best mimics the biophysical behavior of the electric fields; it confines the solution space in the gray matter of the brain and takes into account how the activity in a given area diminishes with the distance from the scalp and thereby assumes smoothness between neighboring sources. The solution space was based on a simplified realistic head model (SMAC; Spinelli et al., 2000) and comprised 3005 points, selected from a 6 mm × 6 mm × 6 mm grid, homogeneously distributed within the gray matter of the average brain of the Montreal Neurological Institute (MNI) that was used as template source space for all subjects. The lead field (or the forward solution) was then solved with an analytical solution with a three-shell spherical head model (brain, skull, and scalp). Numerous experimental and clinical studies have demonstrated that LAURA yields reliable source estimates (Khateb et al., 2007; De Lucia et al., 2010; Plomp et al., 2010; Brodbeck et al., 2011).

Notwithstanding this careful implementation to solve the inverse problem, one is confronted with the problem of thresholding of intracranial sources. There is not and there cannot be a predefined criterion according to which an estimated source can considered as “active.” One way of overcoming this problem of considering absolute estimates of intracranial current density is to perform statistical comparisons between conditions in the source space (James et al., 2008; Britz and Pitts, 2011).

The sources of the ERP were calculated for each subject and each condition previously averaged over the period of interest (i.e., the period showing the significant ERP waveform modulation) and then statistically compared using the same within-subject design as the waveform analysis. To control for multiple comparisons, only significant clusters with a minimal size of 14 consecutive points (*K*_E_) were retained. This spatial criterion was determined with the AlphaSim program.^4^ There was a false positive probability of *p* < 0.005 for observing a cluster of >14 contiguous nodes (see also De Lucia et al., 2010; Knebel and Murray, 2012 for the same approach).

## Results

### Tolerance of tDCS Stimulation

We have evaluated the effects of the stimulation based on a questionnaire proposed by Brunoni et al. (2011). All of the participants tolerated the stimulation well. Participants reported no different effect of scalp tingling sensation at the stimulation site following both anodal and sham tDCS (Mann–Whitney *U*-test, *p* > 0.05). No participant reported adverse effects or asked to interrupt the experiment.

### Behavioral Results

#### Online Tasks

##### Verbal fluency

**FIGURE 2:**
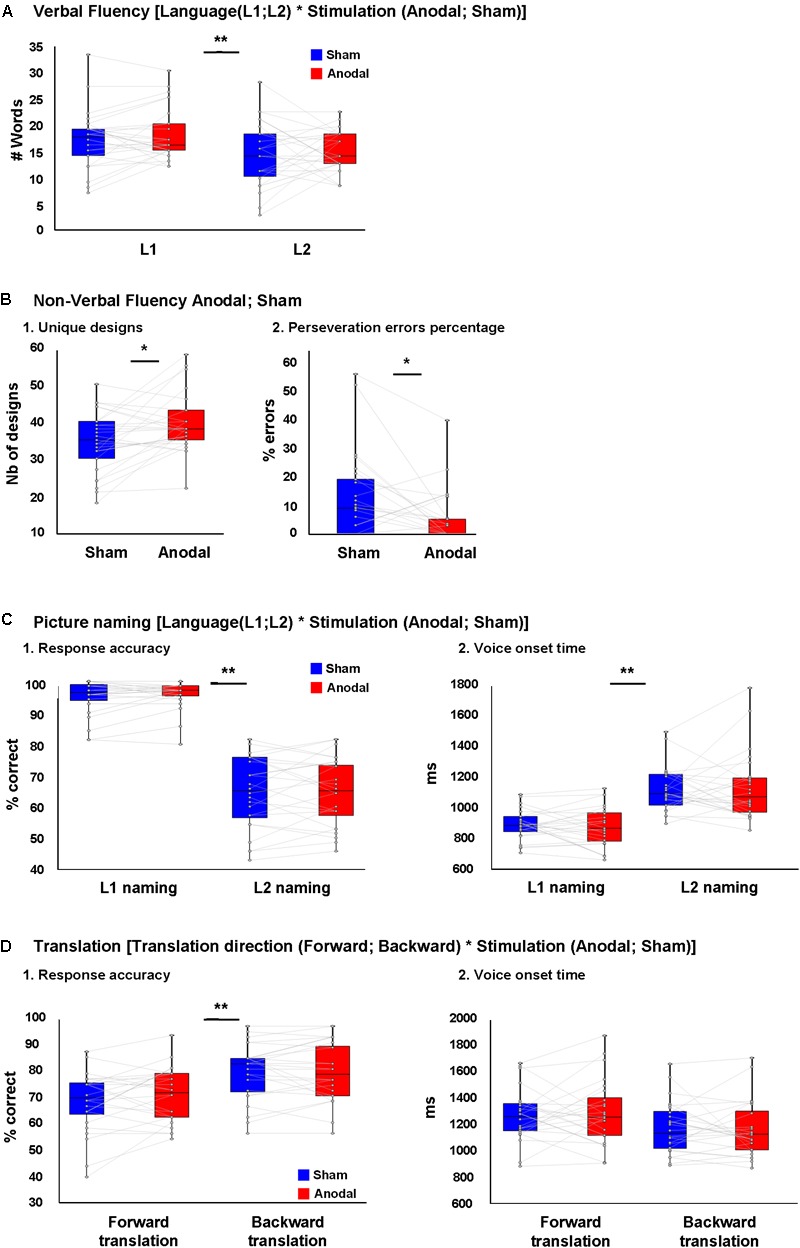
Behavioral results. **(A)** No difference was seen between the number of L1 and L2 words produced during verbal fluency task during anodal or sham-tDCS. **(B)** (1) Higher number of unique designs produced and **(B)** (2) less perseveration error percentages in non-verbal fluency task during anodal-tDCS. **(C)** (1) Response accuracy and **(C)** (2) voice onset time (VOT) in L1 and L2 picture naming following anodal or sham-tDCS showed a main effect of Language (better performance in L1), while no interaction between Language and Stimulation was seen. **(D)** (1) Response accuracy and **(D)** (2) VOT in forward and backward translation following anodal or sham-tDCS showed a main effect of translation direction (better performance in backward translation), while no interaction was seen between translation direction and stimulation. ^∗^*p* < 0.05 and ^∗∗^*p* < 0.01.

**Figure 2A** depicts the results of the verbal fluency task. We found a main effect of Language [*F*(1,25) = 87.1, *p* < 0.001, $\eta_{p}^{2}$ = 0.78], with more words generated in L1 than L2. However, there was no effect of Stimulation [*F*(1,25) = 1.6, *p* = 0.21, $\eta_{p}^{2}$ = 0.061] nor interaction between Language and Stimulation [*F*(1,25) = 0.13, $\eta_{p}^{2}$ = 0.005]. After controlling for L2 proficiency, the main effect of Language [*F*(1,25) = 5.17, *p* = 0.03, $\eta_{p}^{2}$ = 0.17 remained significant with more words were generated in L1 than L2]. However, there was no effect of Stimulation [*F*(1,25) = 1.27, *p* = 0.27, $\eta_{p}^{2}$ = 0.05] nor interaction between Language and Stimulation [*F*(1,25) = 1.23, *p* = 0.78, $\eta_{p}^{2}$ = 0.049].

##### Non-verbal fluency

**Figure 2B** depicts the results of the non-verbal fluency task: there was a higher number of unique designs and a lower percentage of perseverative errors after anodal than sham tDCS [t(25) = 2.76, *p* = 0.011 and t(25) = 2.70, *p* = 0.012, respectively].

#### Offline Tasks

##### Picture naming

**Figure 2C** depicts the behavioral results of the picture naming task. We found a main effect of Language [*F*(1,23) = 220.75, *p* < 0.001, $\eta_{p}^{2}$ = 0.90] with better performance in L1 than L2. However, there was no effect of Stimulation [*F*(1,23) = 0.46, $\eta_{p}^{2}$ = 0.02] nor an interaction between Language and Stimulation [*F*(1,23) = 0.03, $\eta_{p}^{2}$ = 0.001; **Figure 2C**, 1]. After controlling for the effect of L2 proficiency, the effect of Language [*F*(1,23) = 24.75, *p* < 0.001, $\eta_{p}^{2}$ = 0.52] remained significant with better performance in L1 than L2, but there was no effect of Stimulation [*F*(1,23) = 2.45, $\eta_{p}^{2}$ = 0.1] nor an interaction between Language and Stimulation [*F*(1,23) = 0.02, $\eta_{p}^{2}$ = 0.001].

For naming latency (VOT), there was an effect of Language [*F*(1,23) = 70.4, *p* < 0.001, $\eta_{p}^{2}$ = 0.75] with faster latencies in L1 than L2, but no effect of Stimulation [*F*(1,23) < 0.001, $\eta_{p}^{2}$ = 0.001] nor an interaction between Language and Stimulation [*F*(1,23) = 0.46, $\eta_{p}^{2}$ = 0.02; **Figure 2C**, 2]. After controlling for the effect of L2 proficiency, the main effect of Language [*F*(1,23) = 10.99, *p* = 0.003, $\eta_{p}^{2}$ = 0.33] remained significant, but there was no effect of Stimulation [*F*(1,23) = 0.04, $\eta_{p}^{2}$ = 0.002] nor an interaction between Language and Stimulation [*F*(1,23) = 0.172, $\eta_{p}^{2}$ = 0.008].

##### Word translation

**Figure 2D** depicts the behavioral results of the Translation task. For translation performance, there was a main effect of Translation direction [*F*(1,23) = 31.2, *p* < 0.001, $\eta_{p}^{2}$ = 0.58] with backward translation better than forward translation. However, there was no effect of Stimulation [*F*(1,23) = 0.59, $\eta_{p}^{2}$ = 0.02] nor an interaction between Translation and Stimulation [*F*(1,23) = 2.13, *p* = 0.16, $\eta_{p}^{2}$ = 0.08; **Figure 2D**, 1]. After controlling for the effect of L2 proficiency, the main effect of Translation direction no longer reached significance [*F*(1,23) = 3.82, *p* = 0.063, $\eta_{p}^{2}$ = 0.15], and neither the main effect of stimulation, nor the interaction between these factors were significant [*F*(1,23) = 0.19, $\eta_{p}^{2}$ = 0.009 and *F*(1,23) = 0.07, $\eta_{p}^{2}$ = 0.003, respectively].

For translation latency (VOT), there was an effect of Translation direction [*F*(1,23) = 32.7, *p* < 0.001, $\eta_{p}^{2}$ = 0.58] with backward translation faster than forward. However, there were no main effects of Stimulation [*F*(1,23) = 0.047, $\eta_{p}^{2}$ = 0.002] nor significant interaction between Language and Stimulation [*F*(1,23) = 0.87, $\eta_{p}^{2}$ = 0.03; **Figure 2D**, 2]. After controlling for L2 proficiency, the main effect of translation direction failed to reach significance [*F*(1,23) = 3.78, *p* = 0.06, $\eta_{p}^{2}$ = 0.147], and there was no main effect of Stimulation nor a significant interaction between Translation direction and Stimulation [*F*(1,23) = 1.08, $\eta_{p}^{2}$ = 0.047 and *F*(1,23) = 0.046, $\eta_{p}^{2}$ = 0.002, respectively]. We should add that because the data on accuracy in picture naming and translation did not follow normal distribution in some conditions, supplementary analyses were performed on rank transformed picture naming and translation data. This information can be found in Supplementary Material, Presentation 1.

#### Consistency of tDCS Effect Across Tasks

**FIGURE 3:**
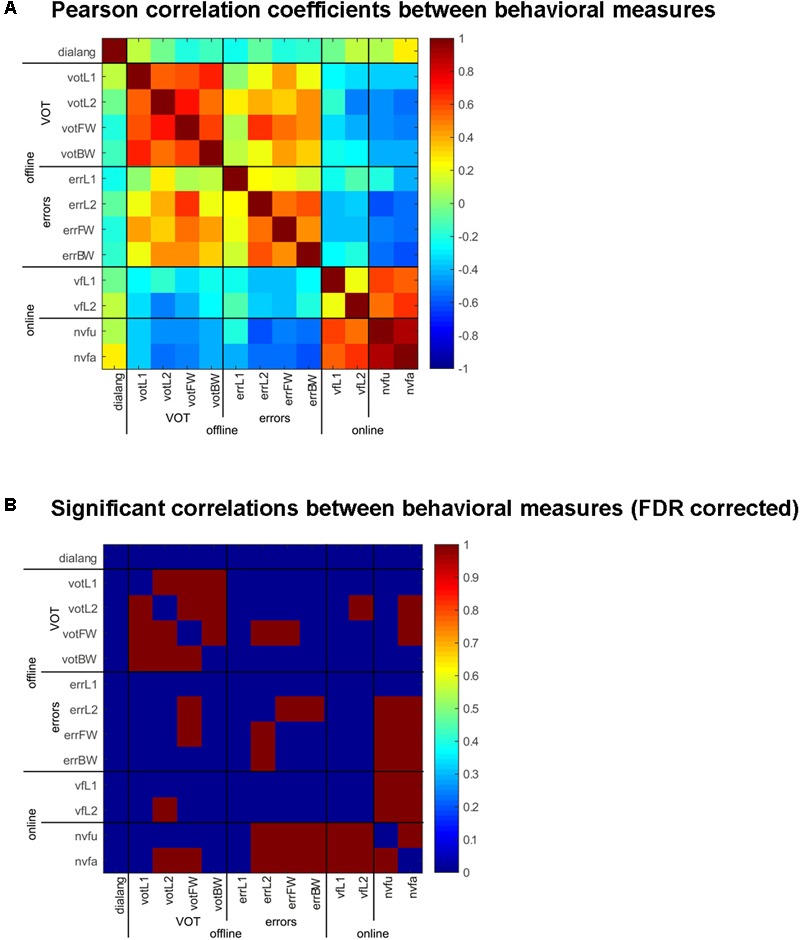
Correlation of behavioral measures in the online and offline tasks and the DIALANG measures. **(A)** Pearson correlation coefficients between the behavioral measures (VOT and errors) in the offline tasks (naming in L1 and L2 and forward and backward translation) and the online tasks [verbal fluency in L1 and L2 and non-verbal fluency (unique and all designs)] comparing sham vs. anodal-tDCS stimulation. Warm colors indicate positive correlations, cold colors indicate negative correlations. **(B)** Significant [FDR-corrected (adjusted *p*-value: *p* = 0.011)] correlations between the behavioral measures are indicated in red, and non-significant values in blue.

Even though there was no overt effect of Stimulation except in the online non-verbal fluency task, tDCS had consistent effects across all tasks, both online and offline: in some subjects, it had facilitating effects and in others it had inhibitory effects, and it did so consistently across all tasks. We computed correlation coefficients between the behavioral measures in the six tasks we employed and the DIALANG score and adjusted the significance level using the false discovery rate; the adjusted *p*-value is 0.0110. These results are depicted in **Figure 3** (Supplementary Figure 1 shows the effects in all tasks for each subject). tDCS had consistent effects in all online tasks, the only ones that did not correlate significantly were verbal fluency in L1 and L2 (see also Supplementary Figure 1). Offline, all measures of VOT (naming in L1 and L2, forward and backward translation) were highly and significantly correlated (Supplementary Figure 1), i.e., subjects were consistently faster or slower in pronouncing words in L1 and L2 after stimulation. A similar effect was obtained for errors: errors committed for naming in L2 were significantly correlated with errors in both forward and backward translation (Supplementary Figure 1), and VOT in forward translation was significantly correlated with errors in naming in L2 and forward translation. Finally, the effects of tDCS in the online non-verbal fluency task was significantly correlated with the online verbal fluency task and with errors committed in all offline tasks as well as with the VOT in naming in L2 and forward translation (the two most difficult conditions, in which words had to be produced in L2).

It is important to note here that the DIALANG vocabulary score did not correlate with any of the behavioral measures, i.e., the consistent effects of tDCS on word production cannot be attributed to language performance measured with the DIALANG vocabulary score.

### Electrical Neuroimaging Results

#### ERP Waveforms

##### Picture naming

**FIGURE 4:**
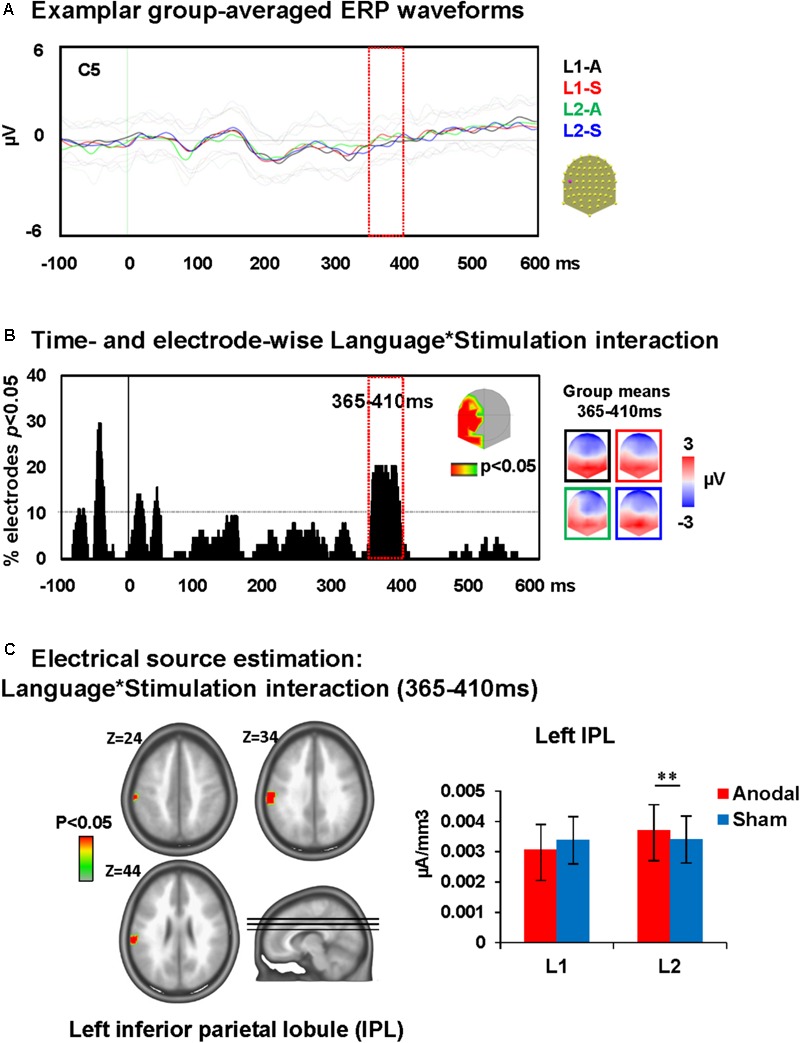
Electrical neuroimaging results: effects of DLPFC tDCS on picture naming ERP modulations. **(A)** Superimposed ERP waveforms for the four conditions of interest (Naming L1-anodal, Naming L1-sham, Naming L2-anodal, and Naming L2-sham) at one exemplary electrode (C5). This figure includes also the standard deviation of the waveforms for each condition (appear in lighter colors). **(B)** Time and electrode-wise statistical analyses of the ERPs. The graph depicts for each post-stimulus time point the percentage of electrodes showing a significant Language × Stimulation interaction (*p* < 0.05). The 365–410 ms period showed a sustained interaction effect (>11 ms for >10% of the electrodes; light orange square). The topographic map represents the electrode sites showing a significant interaction, and on the right the ERP topography for all groups and conditions at the time window of significant interaction [same colors as in **(A)**]. The topographic map of Naming L2-anodal is different from the other three conditions. **(C)** Statistical analysis of the distributed electrical source estimations over the period of significant Language × Stimulation interaction defined in **(C)** (*p* < 0.05; *K*_E_ > 14). *Z* coordinates are indicated on upper left of each MRI slide. ^∗∗^*p* < 0.01.

A time-wise 2 × 2 voltage waveform analysis on all electrodes with the factors Language and Stimulation showed main effects of Language at 110–150 ms on left central and parietal electrodes and 235–380 ms post-stimulus presentation on fronto-central electrodes. There was also an interaction between Language and Stimulation at 365–410 ms on left and posterior electrodes [F(1,23) = 15.8, *p* < 0.001; >11TF]. There was no effect of stimulation on picture naming. The topographic maps for group mean of the ERPs in all conditions showed a different map for picture naming performance in L2 following anodal stimulation when compared to all other conditions (**Figures 4A,B**).

##### Word translation

**FIGURE 5:**
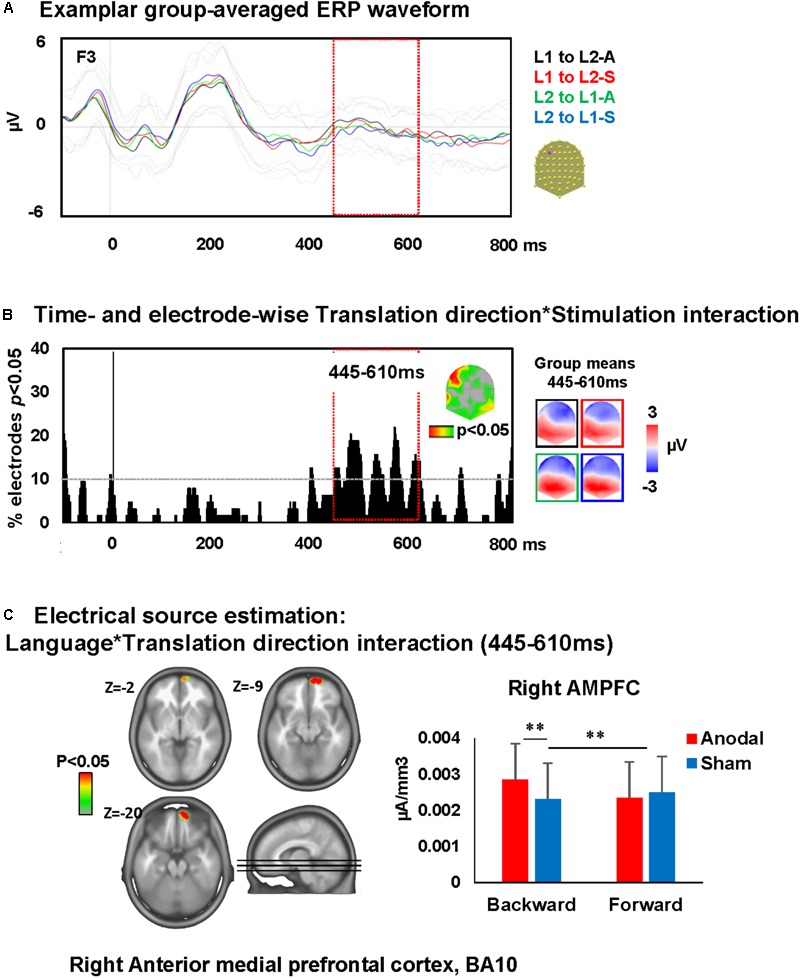
Electrical neuroimaging results: effects of DLPFC tDCS on translation ERP modulations. **(A)** Superimposed ERP waveforms for the four conditions of interest (L1 to L2-anodal, L1 to L2-sham, L2 to L1-anodal, and L2 to L1-sham) at one exemplary electrode (F3). This figure includes also the standard deviation of the waveforms for each condition (appear in lighter colors). **(B)** Time and electrode-wise statistical analyses of the ERPs. The graph depicts for each post-stimulus time point the percentage of electrodes showing a significant Language × Stimulation interaction (*p* < 0.05). The 445–610 ms period showed an interaction effect (>11 ms for >10% of the electrodes; light orange square). The topographic map represents the electrode sites showing a significant interaction, and on the right the ERP topography for all groups and conditions [same colors as in **(A)**]. **(C)** Statistical analysis of the distributed electrical source estimations over the period of significant Language × Stimulation interaction defined in **(C)** (*p* < 0.05; *K*_E_ > 14). *Z* coordinates are indicated on upper left of each MRI slide. ^∗∗^*p* < 0.01.

A time-wise 2 × 2 voltage waveform analysis on all electrodes with Translation direction and Stimulation showed a main effect of Translation direction at 350–600 ms on bilateral fronto-parietal electrodes and 675–750 ms post-stimulus presentation on left and right parietal electrodes and an interaction between Translation direction and Stimulation at 445–610 ms on left frontal electrodes [F(1,23) = 16.6, *p* < 0.001; >11TF]. There was no main effect of stimulation. Visual inspection of the topographic maps for group mean of the ERPs in different conditions suggests a different map for backward translation after anodal stimulation compared to all other conditions (**Figures 5A,B**).

#### Electrical Source Estimation

Repeated measures ANOVA of distributed source estimates for all conditions were performed for each of the 3005 solution points for the period of significant effects identified in voltage waveform analyses, i.e., 365–410 ms for picture naming and 445–610 ms for translation.

##### Picture naming

Analysis revealed that the ERP modulation at around 365–410 ms post-stimulus presentation originated from an interaction between Language and Stimulation [F(1,23) = 4.5, *p* < 0.05, K_E_ > 14 nodes] in the left inferior parietal lobule (IPL; BA 40). This interaction was driven by an increase of activity within the left IPL during picture naming in L2 after anodal stimulation relative to sham stimulation (Dunn, 1961). There were no effects of anodal-tDCS on picture naming in L1 (depicted in **Figure 4C**).

##### Word translation

The ERP modulation at 445–610 ms post-stimulus presentation originated from interactions between Translation direction and Stimulation [*F*(1,23) = 4.3, *p* < 0.05, K_E_ > 14 nodes] in the right anterior medial prefrontal cortex (AMPFC; BA10). This interaction was driven by an increase of activity within the right AMPFC during backward translation after anodal stimulation relative to sham stimulation (*p* < 0.01) and the increase of activity within this area following anodal stimulation in backward but not forward translation (*p* = 0.01). There were no effects of anodal-tDCS on forward translation (depicted in **Figure 5C**).

## Discussion

We investigated the role of EFs in bilingual language production by activating DLPFC by means of anodal-tDCS. To that end, we investigated behavioral performance and ERPs and their sources during picture naming in L1 and L2 and backward and forward translation during the steady tDCS aftereffect. In addition, we investigated verbal and non-verbal fluency during anodal-tDCS stimulation of DLPFC.

According to the neurocognitive model of bilingual language processing (Abutalebi and Green, 2016), it was expected that stimulation of DLPFC using anodal-tDCS would improve fluency measures on a range of tasks. The only effect of anodal-tDCS we found was an improvement in non-verbal fluency during stimulation. Contrary to our expectations, we found no behavioral improvements on verbal fluency during stimulation and no effect on picture naming or word translation tasks following translation. This is in line with evidence summarized in a recent review that shows no effect of tDCS on cognitive performance (Horvath et al., 2015) in healthy individuals. The authors of this study argue that the effects of tDCS are either to weak or obliterated by inter-individual differences. We tackled this issue by assessing the consistency of the effects of tDCS across the six behavioral tasks we used in our study. Even though tDCS does not improve word production in L1 and L2, we can show that it has very consistent effects across all behavioral measures, both online and offline. In a subset of subjects, it has consistently facilitating effects (more drawings produced in the non-verbal fluency task, more words produced in L1 and L2, faster VOT, and less errors in naming in L2 and both forward and backward translation), whereas it has consistently impeding effects in others (less drawings produced in the non-verbal fluency task, less words produced in L1 and L2, slower VOT, and more errors in naming in L2 and both forward and backward translation). Importantly, tDCS only affected L2 production, but not L1 production (apart from VOT). Inter-individual differences in the consistent effect of tDCS have obliterated group effects in the present study. Surprisingly, these inter-individual differences cannot be attributed to L2 performance, at least not to L2 performance measured with the vocabulary subtest of the DIALANG test. An important lesson to be learned from this is that tDCS can have consistently facilitating or inhibitory effects across multiple tasks that are obliterated by inter-individual differences. A solution to this issue is to attempt to elucidate the nature of those consistent effects.

Our EEG source imaging results draw a more differentiated picture of the effects of anodal-tDCS on bilingual language production: we found evidence that anodal-tDCS had a larger effect on L2 picture naming than L1 picture naming at an epoch related to executive functioning between 365 and 410 ms post-stimulus presentation. This was reflected in increased activity in the left IPL (BA 40) after anodal relative to sham stimulation in L2. This is in line with a recent study highlighting the importance of this area in L2 acquisition and performance (Barbeau et al., 2017). In a 12-week intensive language training, activity in this area prior to training predicted success of the training, and activity in area after training correlated with L2 performance.

We also found evidence that anodal-tDCS had a larger effect on backward translation than forward translation at an epoch that is related to phonological retrieval between 445 and 610 ms post-stimulus presentation. This was reflected by increased activity in the right AMPFC (BA 10) in anodal relative to sham stimulation in backward translation. In broad terms, these results are consistent with the results reported by Christoffels et al. (2013) who found larger N400 amplitudes for backward translation.

In the picture naming task, we found a main effect of language on response accuracy and VOT, but no main effect of stimulation and no interaction between language and stimulation. The effect of language is compatible with previous studies (faster and more accurate in L1 than L2; e.g., Kroll and Stewart, 1994; Abutalebi et al., 2008). The EEG results in the picture naming task help to refine this view; even though we do not find an interaction between stimulation and language, we do find this interaction in the ERPs and their concomitant source differences: the effect of anodal-tDCS was bigger than sham stimulation in L2, and no such effects were found in L1. The present findings therefore suggest to us that anodal-tDCS has a specific effect on access to word forms (lexemes) and word form encoding in L2. Note that L2 is assumed to rely more on executive processes that may be reflected here. By means of EEG source localization (LAURA), we determined that the facilitation of L2 picture naming at around 365–410 ms post-stimulus onset is driven by increased activity within the left IPL. Even though we did not find a behavioral effect of anodal-tDCS over DLPFC on picture naming in L2, we can show that stimulating DLPFC recruits left IPL exclusively for L2 production after stimulation in a time window (365–410 ms) important for form encoding (phonological code retrieval, phonological encoding, and syllabification; Indefrey and Levelt, 2004; Indefrey, 2011). While the left IPL is involved in identification and discrimination of syllables (Hickok and Poeppel, 2007), Abutalebi and Green (2007) suggest a specific role for left IPL in bilingual language control, including switching and inhibitory functions. IPL is also known for the integration of semantic information in both language production and comprehension tasks (Geranmayeh et al., 2015).

In the translation task, contrary to our prediction, we did not find a main effect of stimulation nor did we find an interaction between stimulation and translation direction. Like in the picture naming task, our EEG source imaging results shed a more differentiated light onto the underlying brain processes: we found an interaction between translation direction and stimulation in the time window 445–610 ms post-stimulus onset in the ERPs and their concomitant sources. We found increased activity in AMPFC after anodal than sham stimulation in the forward translation condition. AMPFC is recruited more in attention demanding processes as a part of a network guiding cognitive performance (Zysset et al., 2003). Bottini et al. (1994) used PET and showed that the pattern of brain activation in complex language tasks (metaphor comprehension) engages the analog regions in the right hemisphere including right prefrontal cortex.

Our ERP results in the translation task are in line with the results reported by Christoffels et al. (2013) who found that neural processing differed according to the direction of translation at around 200 and 400 ms following stimulus presentation with larger P2 amplitudes for forward translation and larger N400 amplitudes for backward translation. They explained their results as due to the inversed language effect (Meuter and Allport, 1999), which has been interpreted in terms of reduced access to L1 or the inhibition of L1. This may be the result of adaptation to mixed-language situations as a consequence of sustained language control. Translation is a mixed-language situation where access to L1 words is inhibited to facilitate L2 processing, which will result in a higher activation threshold for L1 than for L2. An alternative explanation however is more effortful conceptual processing in backward compared to forward translation. For example, Phillips et al. (2006) report phonological mismatch negativity (PMN) which occurs around 250–350 ms post-stimulus presentation and N400 components amplitude changes in between-language compared to within-language repetitions with English–French bilinguals. The authors argued that these effects (i.e., larger PMN and N400 to the forward translation and larger PMN but minimal N400 activity to the backward translation) indicate a more strongly engaged conceptual processing in backward than forward translation.

The slower RTs in forward relative to backward the translation can be explained in terms of the RHM model (Kroll and Stewart, 1994) by assuming that forward translation in less proficient L2 speakers is mediated *via* the L1 translation equivalent, which then leads to slower responses. In terms of the IC Model (Green, 1998), the results confirm that forward translation is slower and more error prone and associated with inhibition of dominant L1 lexical nodes – and this will be more difficult.

Our behavioral data confirmed longer latencies in the translation compared to the picture naming tasks (around 200 ms longer) suggesting additional processing. This might be related to target lexical selection after semantic processing or control necessary in switching from the input language to the target language. Moreover, retrieval of corresponding phonological codes may be more complex in translation compared to picture naming possibly leading to more processing steps. Thus, although the stimulation effect is seen in time windows that are different to picture naming results, the time period associated with interactions between translation direction and stimulation may also reflect phonological retrieval and form encoding.

Anodal stimulation over DLPFC improved non-verbal fluency performance, i.e., it improved the number of unique designs generated and decreased perseverative errors. Non-verbal fluency is a task that is sensitive to changing demands on executive functioning (Dewing and Battye, 1971). Non-verbal fluency relies on problem-solving to produce unique designs. Robins Wahlin et al. (2015) reported that non-verbal fluency tasks are more sensitive compared to verbal fluency tasks to evaluate cognitive decline in prodromal Huntington disease. The authors suggested that phonemic fluency requires retrieving items stored in the individual’s lexicon, while non-verbal fluency requires imagination of unknown designs not existing in any lexicon. They assume that the less constrained nature of the task may require more EFs. Interestingly, positive effects of neuromodulation on non-verbal fluency are not reported to date. The presence of the effect of anodal-tDCS on non-verbal fluency task can be also explained by performing this task online during tDCS. Previous studies have indeed shown that this approach may be more efficient in inducing behavioral effects as it controls for the changes in strength and consistency of tDCS aftereffects (Romei et al., 2016). One reason for null effects on the verbal fluency may a relatively weak contribution of DLPFC in language-related EF should also be considered. Chouiter et al. (2016) suggested a preferential role of basal ganglia and dorso-lateral temporal areas in phonemic verbal fluency. Because basal ganglia are connected to cortical structures such as DLPFC *via* the ganglia–thalamo-cortical loops, fluency impairment following disruption in ganglia–thalamo-cortical loops has been demonstrated (Chouiter et al., 2016). Our results may be explained by neural specificity and type of fluency tested (left-phoneme generation and right-design generation, e.g., Ruff et al., 1994); left prefrontal cortex has also been shown to influence figure fluency performance.

The observed pattern of the behavioral findings is not in line with the IC model. That is, our behavioral results do not support the role of (DLPFC-mediated) EF in the processing of L2. It is notable that according to the IC model, several brain areas including DLPFC, ACC, and basal ganglia mediate EF in processing language in bilingual brain. Therefore, we are not able to totally rule out the IC model as we have only considered DLPFC mediated executive functioning. However, because the RHM model does not assume the importance of the EF in bilingual language processing, our behavioral findings could be explained by the RHM model.

The absence of behavioral results can be due the weak relative contribution of DLPFC-mediated control processes involved in language production processes even in L2. The lack of sensitivity of discrete behavioral measures to track subtle tDCS-induced changes in brain activity could explain the absence of behavioral results. The selection of stimulation zone can also explain the absence of behavioral improvement in the translation task. Notably, stimulating areas with more language-specific executive control effects [e.g., anterior cingulate cortex (ACC), which has been suggested to be involved in conflict monitoring and response inhibition, especially in translation tasks] could have been more advantageous. However, ACC is rather difficult to be reached and stimulated using transcranial stimulation techniques. Alternatively, the absence of behavioral results in presence of ERP findings can be explained also by applying different neurocognitive strategies to perform the same task (e.g., picture naming). A further alternative explanation would be that, tDCS stimulation in the present study did not produce enough change at neuronal level to become behaviorally measurable (e.g., Cunillera et al., 2016). However, the applied stimulation montage and parameters have been selected following the recommendations of the literature on tDCS stimulation over DLPFC area.

To our knowledge, this is the first study that used the combination of anodal-tDCS over DLPFC and electrical source imaging to assess the role of EF in bilingual language production (picture naming and translation). While we can reliably reproduce behavioral effects of language [picture naming is faster and more accurate in L1 than L2, and backward translation (L2 → L1) is faster and more accurate than forward translation (L1 → L2)], we did not find a main effect of stimulation of DLPFC on bilingual language production nor an interaction between language and stimulation. At first sight, this absence of a stimulation effect is in line with many behavioral studies that failed to find effects of tDCS on cognitive performance (Horvath et al., 2015). However, absence of evidence does not automatically imply evidence of absence. We added a continuous direct measure of brain activity with a high temporal resolution to the discrete behavioral measures which allowed us to elucidate the effects of stimulating DLPFC on bilingual language production: in the picture naming task, we found an interaction between stimulation and language and could show that IPL, an area crucially involved in L2 acquisition and performance is more strongly recruited in L2 after anodal than sham stimulation in a time window related to lexical form encoding. Likewise, in the translation task, we found an interaction between stimulation and translation direction that was absent in the behavioral data: we can show that AMPFC is recruited more strongly during backward than forward translation after anodal than sham stimulation. Continuous measures of brain activity such as EEG and ERPs provide valuable information about how the brain accomplishes a task, and it is important to note that such brain mechanisms do not always have a concomitant behavioral consequence: for example, during shifting and maintenance of visual–spatial attention, alpha power is retinotopically suppressed and enhanced which facilitates the suppression of irrelevant distractors (Rihs et al., 2007, 2009). The fact that this important mechanism underlying the governing of attentional selection has no direct behavioral consequence (alpha power is not related to reaction times and accuracy) does not diminish its relevance. In the language domain, it has been shown that high- and low-working memory span readers show no difference in the comprehension of simple sentences with a thematic violation. However, they differently use animacy and world knowledge to solve the thematic violation which manifests by an N400 for low-span readers and a P600 for high-span readers. Hence, even though there is no behavioral difference in comprehension between the two groups, ERP results provide important information of how comprehension is differentially achieved (Nakano et al., 2010).

In the present study, discrete behavioral measures such as reaction time and accuracy might not be sensitive enough to track the subtle differences induced by anodal-tDCS on L2 production, but our ERP and ERP source imaging results provide more detailed information of how tDCS can affect word production in a second language.

We note that our study has several limitations that encourage further investigation. First, combining tDCS and EEG is and remains a technical challenge and requires solving the trade-off between the rather short-lived tDCS after-effect (∼30 min) and proper electrode preparation. Because it is not possible to completely remove the saline solution used to prepare the tDCS electrodes and to then properly prepare the 64 scalp EEG electrodes, we decided to prepare the EEG montage before the tDCS montage and to then put the remaining EEG electrodes in place. This is not ideal but under the given circumstances the best solution. We used the same electrolyte (NaCl) for both tDCS and EEG electrode preparation to avoid battery potentials and we checked for potential electrode bridges. We can show that the P100 component evoked by the visual stimuli has the canonical waveform, scalp distribution and intracranial sources, which indicates that the tDCS montage did not fundamentally alter the electric field (Supplementary Figure 2). Second, the stimulation would have stronger effect if the target area was selected among the areas which are more specifically involved in language-related EF (e.g., ACC and IPL), although those areas are more difficult to target using tDCS. Third, because of the non-focal nature of tDCS stimulation, one could argue that the observed effect could be due to the effect of stimulation on other cortical areas rather than DLPFC (e.g., the area under the return electrode). This could be investigated using control tDCS montages to compare with used montage in the present study.

Overall, we can learn two positive lessons from the negative behavioral result in our study on anodal-tDCS on word production in the native and a second language: first, it is not the case that anodal-tDCS does not affect word production at all; it rather has strong facilitatory effects in some subjects and strong inhibitory effects in others which is obliterated by considering the effect of stimulation across all subjects. More importantly so, we can show that it has very consistent effects in the six behavioral tasks (two online: verbal and non-verbal fluency, four offline: naming in L1 and L2 and translating into L1 and L2) using two behavioral measures (reaction times and accuracy) in our study. The challenge in future studies is to elucidate the source of such consistent effects. In our case, it would be obvious to attribute these differences to L2 performance, which is not the case: the scores on the DIALANG vocabulary comprehension test do not predict the production of words in a foreign language. Second, using a continuous measure of brain activity with high temporal resolution such as EEG mapping and source analysis appears to be a promising new avenue to study the subtle effects of tDCS on brain and behavior. Before we can make the claim that tDCS has no overt behavioral effect in a task, one should consider the consistency of tDCS across tasks and try to elucidate the nature of inter-individual differences that obliterate its effects on the group level.

## Author Contributions

NR contributed to conception, design, acquisition of data, data analyses, and interpretation of the results and writing of the manuscript. JB contributed to data analyses, interpretation of the results and writing the manuscript. BW contributed to the interpretation of the results and writing the manuscript. KB and J-MA contributed to conception, design, and interpretation of the results. LS contributed to conception of the study and interpretation of the results.

## Conflict of Interest Statement

The authors declare that the research was conducted in the absence of any commercial or financial relationships that could be construed as a potential conflict of interest.

## Footnotes

1 Clinicaltrial.gov

2 http://cartoolcommunity.unige.ch

3 https://zenodo.org/record/1167723#.W0RqEmMVSUl

4 http://afni.nimh.nih.gov/afni
