# Supplementary material for: Dorsolateral Prefrontal Transcranial Direct Current Stimulation Modulates Language Processing but Does Not Facilitate Overt Second Language Word Production
Source: Front Neurosci. 2018 Jul 25;12:490. doi: 10.3389/fnins.2018.00490 (PMC6068342; doi:10.3389/fnins.2018.00490)
Supplement: Supplementary file 1 [file Presentation_1.pdf]

Supplementary analyses:

The data on picture naming and translation accuracy were measured in percentage of correct responses. This data does not follow the normal distribution in some conditions. We have therefore rank transformed the data and performed the analyses on the rank transformed data.

For picture naming, we have implemented the transformed data in a two way ANOVA with factors Language (L1; L2) and Stimulation (Anodal; Sham). The pattern of results was similar to what we have found in the analyses on the raw data: For picture naming accuracy, there was a main effect of Language ($F(1,23)=204.45$, $p<0.001$, $\eta p^2=0.90$; L1 better than L2). However, there was no effect of Stimulation ($F<1$, $\eta p^2<0.001$) nor interaction between Language and Stimulation ($F<1$, $\eta p^2=0.001$).

Rank transformed data on translation accuracy were subjected to a 2 X 2 within subject design with factors Translation direction (Forward; Backward) and Stimulation (Anodal; Sham). The pattern of results was similar to what we have found in the analyses on the raw data: For translation performance, there was a main effect of Translation direction ($F(1,23)= 35.2$, $p<0.001$, $\eta p^2= 0.60$; Backward better than Forward). However, there was no effect of Stimulation ($F(<1$, $\eta p^2= 0.002$) nor interaction between Translation and Stimulation ($F(1,23)=2.25$, $p=0.14$, $\eta p^2= 0.09$).
