# Supplementary figures and images for: Dorsolateral Prefrontal Transcranial Direct Current Stimulation Modulates Language Processing but Does Not Facilitate Overt Second Language Word Production
Source: Front Neurosci. 2018 Jul 25;12:490. doi: 10.3389/fnins.2018.00490 (PMC6068342; doi:10.3389/fnins.2018.00490)

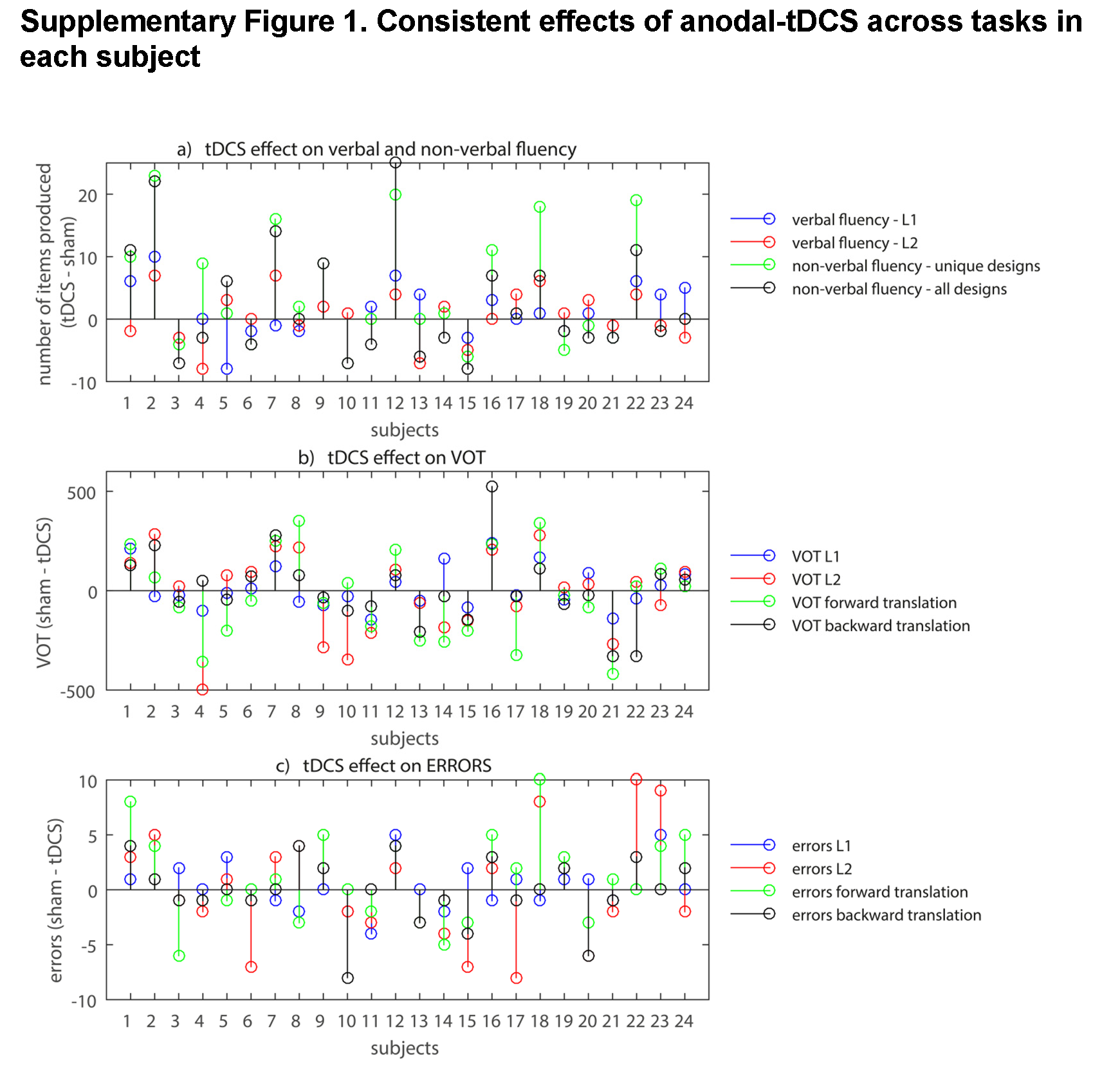

Supplement: Supplementary file 2 [file Image_1.tif]

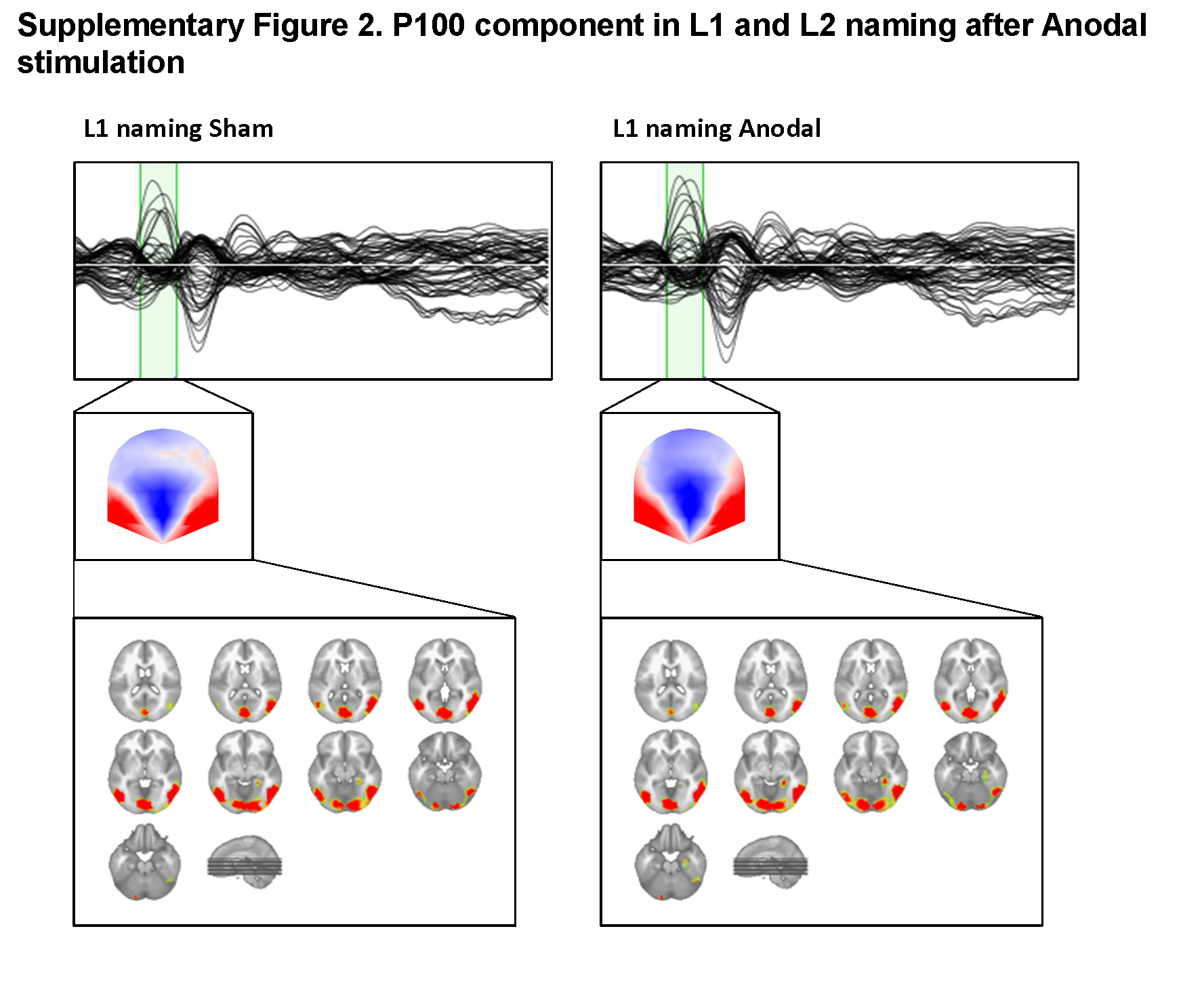

Supplement: Supplementary file 3 [file Image_2.tif]
